# Metabolic syndrome in South Asian immigrants: more than low HDL requiring aggressive management

**DOI:** 10.1186/1476-511X-10-45

**Published:** 2011-03-16

**Authors:** Sunita Dodani, Rebecca Henkhaus, Jo Wick, James Vacek, Kamal Gupta, Lei Dong, Merlin G Butler

**Affiliations:** 1 Center for Post Polio Rehabilitation. 2308 W, 127 street, Leawood, KS 66209, USA; 2 Departments of Psychiatry & Behavioral Sciences and Pediatrics, University of Kansas Medical Center, 3910 Rainbow Blvd, Kansas City, Kansas 66160, USA; 3 Department of Biostatistics, School of Medicine, University of Kansas Medical Center, 3910 Rainbow Blvd, Kansas City, Kansas 66160, USA; 4 Mid America Cardiology, University of Kansas Medical Center, 3910 Rainbow Blvd, Kansas City, Kansas 66160, USA

## Abstract

Aggressive clinical and public health interventions have resulted in significant reduction in coronary artery disease (CAD) worldwide. However, South Asian immigrants (SAIs) exhibit the higher prevalence of CAD and its risk factors as compared with other ethnic populations. The objective of the current study is to assess the prevalence of metabolic syndrome (MS), its association with high density Lipoprotein (HDL) function, Apo lipoprotein *A-I *(*APOA1*) gene polymorphisms, and sub-clinical CAD using common carotid intima-media thickness (CCA-IMT) as a surrogate marker. A community-based cross-sectional study was conducted on SAIs aged 35-65 years. Dysfunctional/pro-inflammatory (Dys-HDL) was determined using novel cell free assay and HDL inflammatory index. Six intronic *APOA1 *gene polymorphisms were analyzed by DNA sequencing. According to the International Diabetes Federation definition, MS prevalence was 29.7% in SAIs without CAD and 26% had HDL inflammatory index ≥ 1 suggesting pro-inflammatory Dys-HDL. Six novel *APOA1 *single nucleotide polymorphisms (SNPs) were analyzed with logistic regression, three SNPs (G2, G3, and G5) were found to be significantly associated with MS (p = 0.039, p = 0.038, p = 0.054). On multi-variate analysis, MS was significantly associated with BMI > 23 (P = 0.005), Apo-A-I levels (p = 0.01), and Lp [a] (p < 0.0001). SAIs are known to be at a disproportionately high risk for CAD that may be attributed to a high burden for MS. There is need to explore and understand non-traditional risk factors with special focus on Dys-HDL, knowing that SAIs have low HDL levels. Large prospective studies are needed to further strengthen current study results.

## Introduction

Despite improvements in clinical outcomes and decrease in event rates by 50% over the past 30 years, coronary artery disease (CAD) continues to be a major cause of death in the US [1]. A disturbing trend toward high rates of CAD, insulin resistance or metabolic syndrome (MS) has been noted among South Asian immigrants (SAIs)- people from the Indian sub-continent (Bangladesh, Pakistan, India, Sri Lanka, Nepal and Bhutan) [2-6]. This is particularly alarming for several reasons; (**i**) South Asians represent one-fifth of the global population. In the US, 3.6 million, or 1.3% of the population, is made up of SAIs [7-10]. SAIs are the fastest growing Asian immigrant population in the US which has more than doubled since the 1980 s (growth rate of 108%), and of that growth, three-fourths is due to immigration [7] and (**ii**) prevalence of CAD in SAIs is twice as high as other immigrant populations [11] and three times higher than in the Framingham Heart Study (FHS), even after adjustment for all conventional risk factors [12-14].

MS plays a causative role in the prevalence of type II diabetes (T2D) as well as premature atherosclerosis in SAIs, a pattern increasingly noted in parallel with migration and urbanization. Current guidelines for the criteria used to define MS including body mass index (BMI) and waist circumference (WC), were predominantly modeled after white Caucasians and are likely to underestimate MS and abdominal obesity in SAIs [13-16]. Evidence suggests that immigration from South Asia to the US, and the acculturation that occurs, exacerbates the consequences of MS and increases CAD risk. Moreover, low HDL is one of the components of MS and SAIs are known to have low HDL. However, assessment of HDL functionality and its correlation with MS is important and has not been studied in any population. In order to understand the type of MS and its association with dysfunctional HDL-Dys-HDL (if present), we conducted a National Institutes of Health (NIH) funded project with an objective to assess the prevalence of MS, its association with CAD and HDL functionality in SAIs. We also assessed Apo lipoprotein A-I (*APOA1) *gene polymorphisms to understand their association with MS and other factors including Dys-HDL and low HDL.

Apo A-I (*APOA1 *gene; Apo A-I protein) is the major protein component of HDL and consists of 243 amino acids, synthesized mainly in the liver and to some extent in the small intestine [17]. The inverse relationship between HDL plasma levels and CAD has been attributed to the role of HDL and its major constituent Apo A and reverse cholesterol transport (RCT). The efficiency of RCT depends on the specific ability of ApoA- Ito promote cellular cholesterol efflux, bind lipids, activate lecithin: cholesterol acyltransferase (LCAT), and form mature HDL that interacts with specific receptors and lipid transfer proteins[18]. The *APOA1 *gene is present along with *APOC3*, *APOA4 *and *APOA5 *genes and located on chromosome 11 (11q23.3-qter) [19]. This gene cluster region contains at least 182 distinct SNPs, making it a relatively heterogeneous genetic region [20]. A recent study of SAIs revealed a prevalence of 6 novel SNPs in the intronic regions of the *APOA1 *gene, one of which (G5: C938T) was significantly associated with low plasma HDL levels [19]. South Asians are commonly known to have low HDL levels and we have shown an association of Apo A-I with low HDL in SAIs [19]. An additional SNP (G-75A) identified in the *APOA1 *gene is located in the 5' untranslated region [20] and is present in 11-35% of the population, with frequency variation depending on ethnicity or geographic origin of the study population [20-22]. We predict that specific *APOA1 *gene polymorphisms may be related to increased MS risk, and therefore increased CAD risk.

Several groups have developed criteria for diagnosing MS; for example, World Health Organization (WHO), National Cholesterol Educational Program ATP III (NCEP ATP III), and International Diabetes Federation (IDF) have proposed different criteria for MS diagnosis (Table 1). Of these, only the IDF considers ethnicity in their criteria [14-16]. Though we present MS prevalence based on all three criteria, we used the IDF guidelines (Appendix 1) for all data analysis in the current study [15,16]. According to the IDF classification, MS is defined as the presence of three or more of the following: 1) increased waist circumference (≥ 90 cm in males, ≥ 80 cm in females), this is an essential component; 2) elevated serum triglycerides (≥150 mg/dl); 3) low HDL cholesterol (< 40 mg/dl in males, <50 mg/dl in females); 4) hypertension (systolic blood pressure ≥130 mmHg and/or diastolic blood pressure ≥85 mmHg) or medical treatment of previously diagnosed hypertension; and 5) elevated fasting glucose levels (≥110 mg/dl). In addition, since lower cut-off values of body mass index (BMI) have been suggested for South Asians (BMI ≥ 23 is considered abnormal), we have used these values for the current study [23].

**Table 1:** Socio-Demographics of Study Group (n = 129)

Variable		n (%)
**Age (years)**		51.30 ± 9.23 *
	Male	51.04 ± 9.64*
	Female	51.68 ± 8.70*
**Gender**		
	Male	76 (58.6)
	Female	53 (41.4)
**Ethnicity**		
	South Indian	33 (25.6)
	Guajarati	23 (17.8)
	Hindi	23 (17.8)
	Bengali	10 (7.8)
	Punjabi	8 (6.2)
	Other	4 (3.2)
**Work Type**		
	Employee full time	91 (70.5)
	Homework	14 (10.9)
	Employee part time	9 (7.0)
	Unemployed	6 (4.7)
	Other	8 (6.2)
**Education**		
	Post-Graduate	67 (51.9)
	Graduate	30 (23.3)
	Undergraduate	24 (18.6)
	Other	1 (0.8)

* Mean ± SD (Standard Deviation)

## Study Design and Methods

Using an epidemiologic cross-sectional study design, SAIs between the ages of 35-65 years without a known history of CAD were randomly recruited from the South and Midwest regions of the US (States of Georgia, Kansas and Missouri). We chose this age range because CAD and its risk factors occur at younger ages in SAIs as compared to other populations [3]. The SAI population in the US is most readily accessed through their temples of worship. Therefore, although study information was made available and distributed using different methods, the majority of study subject recruitment was done through Hindu temples. This approach was used as no national level census or data are available on South Asians providing a correct estimate of total population. Therefore, we understand that results of this study may not be generalizable; however, most SAIs visit temples on weekends representing several ethnic groups. Study information was made available by distributing flyers in the temples and announcements through local newspapers outlining the purpose, rationale, and design of the study. After the written informed consent was obtained, information on socio-demographic status, ethnicity (based on spoken language), personal lifestyle characteristics, and both traditional and non-traditional risk factors for CAD were obtained. Twelve-hour fasting blood samples were collected for measurements of high sensitivity C-reactive protein (hsCRP), total lipid testing including total cholesterol, Triglycerides (TGs), high density lipoprotein (HDL), low density lipoprotein (LDL), and lipoprotein a (Lp[a]). Insulin, Fibrinogen, Homocysteine and Apo A-I serum levels were also measured.

### Carotid Ultrasound Doppler for Common Carotid Intima-Media Thickness (CCA-IMT)

CCA-IMT is defined by Pingoli and colleagues as the distance from the leading edge of the lumen-intima interface of the far wall to the leading edge of the media -adventitia interface of the far wall [24]. B-mode ultrasound scanning of bilateral CCAs was performed by a trained non-invasive vascular ultrasound technician at the University of Kansas Medical center study clinics, using SonoSite MicroMaxx™ ultrasound machine (SonoSite, Inc Bothell, WA) with a 10.0 MHz linear array transducer. Both CCAs were scanned in supine position. A total of eight images were obtained (four on each side), 1 cm proximal to the carotid bulb using a posterior wall (far wall) approach. ECG leads were placed to obtain end-diastolic measurements. Images were recorded and stored on a disk. The CCA-IMT approach for IMT measurements was preferred because the CCA-IMT is reproducible and predictive of future cardiovascular events, and data collection is more complete than the use of other non-invasive markers [25,26]. Measurements of the internal carotid and bifurcation segments tend to have many more missing values. The Mannheim Intima-Media Thickness Consensus suggested that measurement of the CCA is ideal [25].

Any focal thickening of the intima-media complex or carotid plaque was not included in the analysis. Two cardiologists, who were blinded to participants' identities and clinical information, analyzed stored images by using the SonoCalc™ IMT software. Measurement of the far wall of the carotid artery was preferred, since studies comparing ultrasound measurements with histology suggest that far-wall CCA-IMT measurements are more indicative of the true thickness of the arterial wall [26,27]. Near-wall CCA measurements, in comparison, are limited by their dependence on the axial resolution, gain settings of the equipment used and in addition, show greater variation between repeated measurements [25]. Participants with values equal to or greater than 0.80 mm were considered to be IMT positive. Previous epidemiological studies suggest that a value of IMT at or above 0.80 mm is associated with a significantly increased absolute risk of CAD [26]. In this study CCA-IMT values of 0.80 mm or more were considered abnormal. CCA-IMT values were adjusted for age as age can influence IMT [25,26]. We did not include plaque readings in this study.

#### Assessment of Dysfunctional HDL (Dys-HDL)

The diagnosis of Dys-HDL has historically been made with a cell-based assay that requires endothelial cells, smooth muscle cells, and monocytes. However, the use of a cell-based assay is not practical for large-scale studies. A cell-free assay has been developed to detect HDL that is dysfunctional [28]. The details on Dys-HDL assessment using the cell-free assay have been published previously [28,29]. Briefly, this is a rapid test for HDL function that does not require cells and gives results highly comparable to those of the previously described cell-based assay. HDL was isolated from blood samples using dextran sulphate precipitation.LDL, necessary in the cell-free assay for testing the ability of HDL to protect against LDL oxidation, was prepared from a normal donor, aliquoted and then cryo-preserved in sucrose. Dichlorofluorescein-diacetate (DCFH-DA) was dissolved in fresh methanol at 2.0 mg/ml, incubated at room temperature, and protected from light for 30 min, which resulted in the release of dichlorofluorescein (DCFH) producing an intense fluorescence upon interaction with oxidized lipid. Fluorescence was determined using a plate reader (Spectra Max, Gemini XS; Molecular Devices) at an excitation wavelength of 485 nm, an emission wavelength of 530 nm, and a cutoff of 515 nm with the photomultiplier sensitivity set at medium. For this study, the coefficient of variation for this assay was 9.6% [28]. Similarly, the HDL-inflammatory index (HII) was calculated by normalizing the cell-free assay values obtained for LDL alone as 1.0 [29]. If the addition of a test HDL resulted in a value of 1.0 or greater, the test HDL was classified as pro-inflammatory (dysfunctional). Conversely, if the addition of the standard normal LDL together with a test HDL resulted in a value less than 1.0, the test HDL was classified as anti-inflammatory. To support the results of the cell free assay, we also measured serum hsCRP.

#### DNA Extraction From Blood Specimens

Each blood sample was assigned a unique DNA identification code. Genomic DNA was extracted from whole blood (2 mls) using the Qiagen DNA Isolation Kit (Qiagen^®^, Valencia, CA, USA), according to the manufacturer's protocol, which yields 4-12 μg of high quality DNA. An aliquot of DNA was diluted and the absorbance at λ 260 nm and λ 280 nm measured using an Eppendorf Biophotometer for verification of quality and concentration. DNA samples were diluted to 50 ng/μl and stored at -20°C.

#### DNA Sequencing of *APOA1 *Gene

PCR amplification was performed on genomic DNA to amplify a 1683 bp fragment of the *APOA1 *gene encompassing the SNPs to be analyzed using forward 5' CACAATGGACAATGGCAACT 3' and reverse 5' CCAGATCCTTGCTCATCTCC 3' PCR primers. The PCR fragment was purified using the QIAquick PCR purification kit (Qiagen, Valencia, CA). Sequencing was performed commercially by ACGT Inc. (Wheeling, IL) using a series of two sequencing primers on the forward strand (first sequencing primer: 5' CTTGACCCCTGCCCTGCAGC 3'; second sequencing primer: 5' CGGCAGAGACTATGTGTCCCAG 3') which completely encompassed the six SNPs within a range of high fidelity sequencing (< 600 bp). Sequencing data for each subject were provided in the form of raw electropherogram files from which DNA sequences were derived using Ridom Trace Edit software (Ridom Bioinformatics, Würzburg, Germany). All SNPs were determined and verified both from the derived DNA sequence and visual inspection of the electropherogram. Results were analyzed by comparative NCBI BLAST sequence analysis. The reference sequence used was NCBI RefSeq NC_000011.9, derived from the Genome Reference Consortium Human Build 37 (GRCh37), Primary Assembly http://www.ncbi.nlm.nih.gov/gene.

### Power Calculations and Statistical Analysis

We enrolled 148 first generation SAIs with different ethnic backgrounds. This is a pilot study survey with a fixed sample of 129 to assess the MS and CAD risk factor profile in SAIs. The power calculation for assessing dysfunctional HDL were based on a chi-square contingency table analysis [Dys- HDL (Yes/No) vs. MS (Yes/No)], from available data on MS in South Asians [22]. Assuming that 15% of the 129 subjects have MS using IDF criteria and 5% among non-MS, we have a 91% power at 5% alpha level and 85% power at 1% alpha level to detect the Dys-HDL difference in two groups.

Baseline socio-demographic characteristics and CAD risk factors were summarized by frequency distributions and percentages for qualitative measures and means and standard deviations for quantitative measures. Figures were presented in percent estimates. Maximum likelihood estimates and asymptotic 95% confidence intervals were calculated for the prevalence of disease/diagnosis outcome measures. Bivariate tests of association and odds ratios were performed by the Fisher Exact Test methods with age and gender adjustment where necessary. Genetic analysis of *APO A1 *were analyzed using logic regression methods. All statistical tests were two-sided and performed at the 0.05 level of significance.

## Results

Of the total sample of 148 subjects, complete information was obtained on 129 subjects, constituting our study sample (Table 1). The mean age of subjects was 51 ± 9.23 years with almost an equal number of males and females (Table 1). The study group presented a homogenous mixture of various ethnicities including Hindi speaking (18%), Gujratis (18%), and South Indians (26%). More than 50% received up to post-graduate level education. CAD risk factors prevalence (Table 2) was (**a**) hypertension-45% (**b**) high cholesterol ≥ 200 mg/dl -41.6%, (**c**) HDL < 40 mg/dl-26.4% (**d**) LDL ≥ 150 mg/dl-16.9%, (**e**) Lp[a]-35.7% (**f**) hsCRP (≥ 5)-48.7%, (**g**) BMI ≥ 23-78.4% (**h**) obesity (BMI ≥ 30)-18.2%; (**i**) family history of CAD and T2 D was 34.4% and 48.4%, respectively. Eighty-three percent of our subjects were physically active. Sub-clinical CAD using CCA-IMT ≥ 0.8 mm (as a surrogate marker) was seen in 38.5% of subjects. Increased obesity is also reflected by an increased waist circumferences in both genders in this study (Table 3).

**Table 2:** CAD Risk Factors and Other Markers in Study Group (n = 129)

Variables		n (%)	Mean ± SD
**BMI**			26.37 ± 5.08
	Normal (< 23)	27(21.62)	21.84 ± 1.68
	Overweight (23 - 30)	76(60.14)	25.82 ± 2.05
	Obese (≥ 30)	22 (18.24)	34.07 ± 6.45
**Total LDL**			117.63 ± 35.61
	Normal (< 150 mg/dl)	103(83.06)	106.08 ± 24.73
	Abnormal (≥ 150 mg/dl)	21(16.94)	174.95 ± 24.44
**Total HDL**			48.38 ± 10.99
	Normal (> 40 mg/dl)	92(73.6)	52.95 ± 8.98
	Abnormal (≤ 40 mg/dl)	33(26.4)	35.79 ± 4.31
**HDL2 ^¥^**			12.03 ± 3.69
	Normal (≥10 mg/dl)	26(34.67)	8.19 ± 1.81
	Abnormal (< 10 mg/dl)	49(65.33)	14.12 ± 2.63
**HDL3^%^**			35.91 ±8.25
	Normal (≥30 mg/dl)	18(24.0)	26.11 ± 3.34
	Abnormal (< 30 mg/dl)	57(76.0)	39.02 ± 6.86
Dys-HDL*			0.83 ± 0.74
	Normal (< 1.0)	88(73.95)	0.53 ± 0.17
	Dysfunctional (≥ 1.0)	31(26.05)	1.71 ± 1.02
**Total Cholesterol**			193.17 ± 38.97
	Normal (< 200 mg/dl)	73(58.4)	167.74 ± 22.34
	Abnormal (≥ 200 mg/dl)	52(41.6)	229.31 ± 27.37
**Triglycerides**			160.44 ± 114.56
	Normal (< 150 mg/dl)	73(58.4)	99.23 ± 26.88
	Abnormal (≥ 150 mg/dl)	52(41.6)	246.90 ± 134.70
**Lipoprotein [a]**			13.61 ± 18.99
	Normal (< 10 mg/dl)	79(64.23)	4.59 ± 1.79
	Abnormal (≥10 mg/dl)	44(35.77)	30.02 ± 24.46
**Apo lipoprotein A-I**			150.36 ± 31.94
	Normal (94 - 176 mg/dl)	95(76.0)	142.06 ± 22.19
	Abnormal (else)	30(24.0)	178.13 ± 41.30
hsCRP**			3.32 ± 2.56
	Normal (< 5 mg/L)	63(51.22)	1.24 ± 1.09
	Abnormal (≥ 5 mg/L)	60(48.78)	5.55 ± 1.60
**Homocysteine**			10.34 ± 7.71
	Normal (< 12 umol/L)	74(77.89)	7.96 ± 2.06
	Abnormal (≥ 12 umol/L)	21(22.11)	18.79 ± 13.08
**CCA-IMT**			0.73 ± 0.17
	Normal (< 0.8 mm)	48(61.54)	0.649 ± 0.094
	Abnormal (≥ 0.8 mm)	30(38.46)	0.916 ± 0.15
**Waist Circumference (cm)**			93.72 ±14.08
	Male	61 (48.66)	95.53 ± 12.74
	Female	46 (27.98)	91.47 ± 15.58
**Physical Activity**	No	20 (15.50)	
	Yes	109 (84.50)	
**Smoking**	No	121 (93.80)	
	Yes	8 (6.20)	
**Type II Diabetes^#^**	No	89 (69.53)	
	Yes	39 (30.47)	
**High Blood Pressure^#^**	No	70 (54.69)	
	Yes	58 (45.31)	
**Family history of T2D^$^**	No	48 (43.24)	
	Yes	63 (56.76)	
**Family history of CAD^$^**	No	67 (60.36)	
	Yes	44 (39.64)	

*Dys-HDL = Dysfunctional HDL measure by HDL Inflammatory index; **hsCRP = High sensitivity C reactive protein; ^# ^History/examination and/or blood test; ^$ ^T2D = Type II diabetes; CAD = Coronary Artery Disease.

**Table 3:** Prevalence of Metabolic Syndrome (MS)

		n	%
**MS by IDF**	**NO**	68	53.1
	
	**YES**	38	29.7

**MS by NCEP**	**NO**	76	59.4
	
	**YES**	52	40.6

**MS by EUROPEAN GROUP**	**NO**	96	75.0
	
	**YES**	32	25.0

**MS by WHO**	**NO**	111	86.7
	
	**YES**	17	13.3

European group = European Group for the Study of Insulin Resistance; IDF = International Diabetes Federation;

NCEP ATP III = National Cholesterol Education Program Adult Treatment Panel.

### MS Prevalence and Associations with Risk Factors

**Figure 1:**
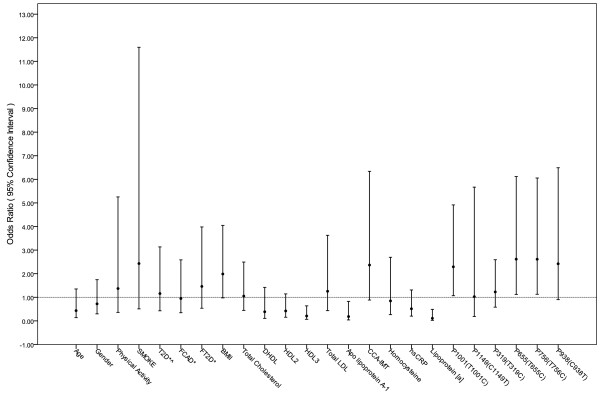
Odds Ratio and 95% Confidence Interval for MS with CAD's risk factors and Apo A-1 SNPs * T2D = type 2 diabetes; CAD = Coronary Artery Disease; FT2D = Family History of T2D; FCAD = Family History of CAD;BMI = Body Mass Index; CCA-IMT = Common carotid artery intimae media thickness; HII = HDL Inflammatory index measured by using cell-free assay; hsCRP = high sensitivity C Reactive Protein; ^History/examination and/or blood test.

Based on the IDF definition, MS was seen in 29.7% of SAIs without CAD. Whereas according to WHO and NCEP ATP III criteria, the prevalence of MS was 13.3% and 40%, respectively, indicating under- and over-estimation of MS prevalence by WHO and NCEP ATP III criteria, respectively (Table 2). On multi-variate analysis using Fisher Exact test, MS was significantly associated with BMI > 23 (p < 0.005), Apo-A-I levels (p < 0.01), and Lp[a] (p < 0.0001) (Table 4, Figure 1).

**Table 4:** Association of CAD risk factors with MS (n = 129)

		MS		P-Value**
			
Variable		No	Yes	
**Age (years)**	< 40	9 (8.0)+	6 (5.3)	0.128
	≥ 40	76 (67.3)	22 (19.5)	
**Gender**	Male	48 (42.5)	18 (15.9)	0.308
	Female	37 (32.7)	10 (8.8)	
**Physical Activity**	No	12 (10.6)	3 (2.7)	0.461
	Yes	73 (64.6)	25 (22.1)	
**Smoke**	No	81 (71.7)	25 (22.1)	0.234
	Yes	4 (3.5)	3 (2.7)	
**Type 2 Diabetes^#^**	No	66 (58.4)	21 (18.6)	0.479
	Yes	19 (16.8)	6 (6.2)	
**Family history of T2D^$^**	No	37 (38.9)	8 (8.4)	0.313
	Yes	38 (40.0)	12 (12.6)	
**Family history of CAD^$^**	No	44 (46.3)	12 (12.6)	0.326
	Yes	31 (32.6)	8 (8.4)	
**Body Mass Index (kg/m^2)**	≤23	22 (20.0)	3 (2.7)	**0.005**
	23 - 30	48 (43.6)	18 (16.4)	
	≥30	12 (10.9)	7 (6.4)	
**Total LDL**	Normal (< 150 mg/dl)	69 (61.6)	22 (19.6)	0.433
	Abnormal (≥ 150 mg/dl)	15 (13.4)	6 (5.4)	
**Total Cholesterol**	Normal (< 200 mg/dl)	49 (43.8)	16 (14.3)	0.542
	Abnormal (≥ 200 mg/dl)	35 (31.3)	12 (10.7)	
**Apo lipoprotein A-1**	Normal (94 - 176 mg/dl)	59 (52.7)	26 (23.2)	**0.011**
	Abnormal	25 (22.3)	2 (1.8)	
**Lipoprotein [a]**	Normal (< 10 mg/dl)	48 (43.6)	26 (23.6)	**< 0.0001**
	Abnormal (≥10 mg/dl)	34 (30.9)	2 (1.8)	
hsCRP*	Normal (< 5 mg/L)	45 (40.9)	18 (16.4)	0.118
	Abnormal (≥ 5 mg/L)	39 (35.5)	8 (7.3)	
**Homocysteine**	Normal (< 12 umol/L)	44 (53.7)	20 (24.4)	0.512
	Abnormal (≥ 12 umol/L)	13 (15.9)	5 (6.1)	

^+ ^n (%); *hsCRP = high sensitivity C reactive protein; ^# ^History/examination and/or blood test;

^$ ^T2D = type II diabetes; ^$^CAD = Coronary Artery Disease; ** Fisher's Exact test;

### Dys-HDL, Low HDL and *APOA1 *Gene Polymorphisms

Dys-HDL was measured by HDL inflammatory index using a novel cell free assay [28]. Twenty-six percent of our subjects had an HDL inflammatory index ≥ 1 suggesting pro-inflammatory HDL (Dys-HDL). Six *APOA1 *single nucleotide polymorphisms (SNPs) (Table 5) were analyzed discovered in the SAI in this study. These *APOA1 *polymorphisms were also observed in previous studies [19]. On logistic regression analysis, three of the six SNPs (G2, G3, and G5) were found to be significantly associated with MS and may be related to high MS prevalence in SAIs. On the Fisher Exact test analysis, the association of MS with Dys-HDL (p = 0.0348) and CCA-IMT (p = 0.0024) was statistically significant even after age and gender adjustment (Table 6). The odds ratio with 95% confidence interval of CAD risk factors and *APOA1 *SNPs with MS are shown in Figure 1.

**Table 5:** *APOA1 *SNPs Association with Metabolic Syndrome (n = 94)

*APOA1 *Gene Polymorphisms		Metabolic Syndrome	
		
		Absent	Present
**G1 P319(T319C)**	WT	36 (42.5%)	13 (15.3%)
	Heterozygous	25 (29.4%)	4 (4.7%)
	Mutant	3 (3.5%)	4 (4.7%)
	P-value^$^	0.940	

**G2 P655(T655C)**	WT	12 (14.1%)	0 (0.0%)
	Heterozygous	22 (25.9%)	7 (8.2%)
	Mutant	29 (34.1%)	15 (17.7%)
	P-value	**0.040**	

**G3 P756(T756C)**	WT	12 (14.0%)	0 (0.0%)
	Heterozygous	21 (24.4%)	7 (8.1%)
	Mutant	30 (34.9%)	16 (18.6%)
	P-value	**0.039**	

**G4 P938(C938T)**	WT	51 (59.3%)	15 (17.4%)
	Heterozygous	12 (14.0%)	7 (8.1%)
	Mutant	0 (0.0%)	1 (1.2%)
	P-value	0.109	

**G5 P1001(T1001C)**	WT	12 (13.5%)	0 (0.0%)
	Heterozygous	27 (30.3%)	11 (12.4%)
	Mutant	25 (28.1%)	15 (16.9%)
	P-value	**0.054**	

**G6 P1149(C1149T)**	WT	59 (66.3%)	23 (25.8%)
	Heterozygous	5 (5.6%)	2 (2.3%)
	Mutant	0 (0.0%)	0 (0.0%)
	P-value	0.976	

*APOA1 *= Apo lipoprotein A 1 gene, WT = Wild Type; ^$ ^P-values from the logistical regression analysis

**Table 6:** Association of MS, Dys-HDL and CCA-IMT ((age and gender adjusted) (n = 129)

Variable		CCA-IMT		**P-Value**^$^
			
		< 0.8 mm	≥0.8 mm	
**MS**	**No**	46 (55.42)^#^	15 (18.07)	**0.0348**
	**Yes**	11 (13.25)	11 (13.25)	

**€y€-HDL**	**HDL index <1**	48 (51.61)	16 (17.20)	**0.0024**
	**HDL index ≥1**	12 (12.90)	17 (18.28)	

CCA-IMT = Common carotid artery intima media thickness; MS = Metabolic Syndrome, defined according to IDF Central obesity and two or more of fasting glucose (≥100 mg/dl or T2D), obesity, HDL (≥40 mg/dl), Blood Pressure (≥130/≥85 mmHg); and Triglycerides (≥150 mg/d); Dys-HDL = Dysfunctional HDL measure by HDL Inflammatory index using cell free assay

^$ ^Fisher's Exact Test

## Discussions

It is predicted that more than one-half the world's CVD burden will be borne by the people from the Indian subcontinent in the next decade [30]. There is evidence to suggest that the presence of MS predicts the future risk for T2 D and CAD. MS is a common and complex disorder combining obesity, dyslipidemia, hypertension, and insulin resistance. It is a primary risk factor for T2 D and CVD. In this study, we showed for the first time that MS is associated with Apo-A-I levels as well as *APOA1 *gene polymorphisms. This may relate to low HDL and/or Dys-HDL in SAIs predisposing them to increased future risk of CAD. However, larger studies are required to confirm these findings. Interestingly, the conventional descriptions of MS have no mention of genetic factors serving as CAD risk markers.

The most accepted and unifying hypothesis to describe the patho-physiology of the MS is insulin resistance. Our study also supports that SAIs have high T2 D prevalence rates despite low body weight (i.e. 17% in this study versus 8% in Caucasians) [4]. The majority of SAIs have a smaller body habitus. Obesity, by its classic definition, is rare among SAIs [11,15,16] as seen in this study, as well. However, the peculiar body habitus and in particular, the unique pattern of fat distribution, are critically significant risk factors described specifically for SAIs. Moreover, SAIs have a higher visceral fat mass than persons of other ethnicities with comparable BMI [4-7,15,27]. The fat distribution is focused in the abdominal visceral space, and this peculiarity is seen in subjects of normal or even subnormal body weight. Our study findings also support the fact that though BMI was not significantly high in this group, increased visceral adiposity was reflected in increased waist circumference in both males and female SAIs (Table 2). This factor has a direct relationship to an increased risk of CAD [12]. Accordingly, and correctly so, the description of BMI is corrected for the SAIs race [16]. Thus obesity, by its classic definition, is not a metabolic risk factor in the SAIs. In defining MS unique to the SAIs, it is important to recognize the accelerated progression of coronary atherosclerosis, leading to CAD at a younger age [3]. The distinct features of MS with accelerated atherosclerosis in the SAIs include visceral adiposity, insulin resistance/hyperinsulinaemia, high LP(a) levels, low HDL levels, and abnormal Apo A-I levels (Table 4). Although prevalence of MS and dyslipidemia is determined by interplay between environment and genetic factors, family and twin studies have demonstrated a strong genetic heritability, accounting for up to 66% of the variability in HDL and Apo A-I levels [8]. Several studies in Caucasian, SAIs, and other ethnic groups have shown that specific lipoprotein polymorphisms do play a role and are considered predictors of MS [31-34]. *APOA1 *gene polymorphisms have been implicated to explain the variability in HDL levels as well as to its dysfunction [31-35]. Furthermore, our findings involving novel SNPs in *APOA1 *gene in SAIs [19] open avenues for further study of SNPs in a larger sample and in relation to other conventional and non-conventional CAD risk factors, including MS.

The INTERHEART study reported a gradual increase in the incidence of traditional risk factors for CAD in the Indian Subcontinent [3]. Though this observational study failed to establish a causative effect, this development coupled with current study findings, in general and genetic polymorphisms related to MS (considered as an intermediate outcome for CAD) in particular, can create an explosive situation in the SAI communities, posing the threat of an unbridled and unprecedented spread of CAD. This underlines the urgent need to act quickly.

Similarly, in this cross sectional study of SAIs, we found a significant relationship for MS with abnormal ApoA-I levels, Lp [a] and importantly three of six SNPs of the *APOA1*gene. These results support our hypothesis of a possible pivotal role for *APOA1 *gene variation predicting higher prevalence of MS and possibly CAD. All six SNPs that were analyzed are intronic or located in the non-coding region of the *APOA1 *gene. The intron was historically thought to be a non-essential region of DNA; however, with advancement in molecular biology, intronic regions are now known to play a role in the regulation of transcription and translational processes or in linkage disequilibrium with other causal mutations.

The ApoA-I protein is essential for maintaining the antioxidant and RCT (reverse cholesterol transport) function of HDL. Decreased HDL is a well known risk factor and predictor for CAD [2-6]. The growing body of research looking into the qualitative assays of HDL, etiology of Dys-HDL and its role in the pathogenesis of atherosclerosis will eventually explain the high CAD risk in a patient with normal HDL. Dys-HDL is not only ineffective as an antioxidant but, paradoxically, appears to be pro-oxidant by promoting LDL oxidation and monocyte chemo-tactic activity in the human artery wall as well as increasing HDL lipid hydroperoxides, as assessed by its lipid peroxide content [33]. This pro-inflammatory dysfunctional HDL accumulates oxidants that inhibit HDL-associated antioxidant enzymes, myeloperoxidases. This results in Apo A-I oxidation that eventually blocks the function of ApoA-1 and promotes cholesterol efflux, consequently promoting the formation of LDL derived oxidized lipids [34,35]. The mechanisms underlying this phenomenon are not completely understood but it is thought that Apo A-I variants are susceptible to oxidation and nitration [34]. The presence of Dys-HDL may substantiate the CAD risk in MS patients at the onset of T2D; however, additional studies are needed to provide more supportive data.

Several limitations of this study must be considered. First, this was a cross-sectional pilot study and, as in all such studies, the data are exploratory; they do not allow the establishment of causality and do not account for changes over time. Second, we recruited participants from local

Hindu temples, and therefore participants may not be completely representative of the South Asian community. However, people attending these temples were from mixed ethnic backgrounds, and data were collected from participants who attended weekend worship services, which in general are attended by South Asians from different and diverse ethnic groups. Therefore, we anticipate that the selection bias is minimal and the sample is representative of SAIs living in the US. On the same token, we also understand the heterogeneity among South Asians, and more research is warranted in each ethnic group. Though these ethnic groups are from South Asians, among them prevalence of MS and its determinants may differ Third, we did not assess diet in this study and is an area of further work in future studies. Last, we were not able to undertake further testing, and since this was a small sample size, we were not able to make definitive conclusions regarding the association of dysfunctional HDL with CAD and its risk factors. A larger longitudinal study has been proposed.

## Conclusion

Several studies have shown that SAIs have a higher prevalence of CAD and MS (as per IDF criteria) compared to other ethnic groups. In fact, evidence has shown that MS is twice as prevalent in SAIs living in the US compared to native Caucasians (30% versus 13%) [8]. Our study showed that three of the six novel *APOA1 *SNPs were significantly associated with MS, substantiating the CAD risk for *APOA1 *gene variants in SAIs. Though larger cohort studies are required to establish the etiologic role of SNPs in MS as well as of Dys-HDL, their identification can be obtained at an earlier age and aggressive measures, especially targeting HDL therapies may prevent the future risk of CAD and clustering of risk factors [36]. Treating Dys-HDL with statins and niacin therapy, particularly in SAIs is important to reduce the excess CAD risk in this high risk group. We propose that the screening of Dys-HDL in addition to conventional risk factors in high risk groups is a potential target for prevention of cardiovascular disease.

## Abbreviations

Apo A-I: Apo lipoprotein A-I; *APOA1*= Apo lipoprotein A-I gene; BMI: Body mass index; CAD: Coronary artery disease; CCA-IMT: Common Carotid Intima-Media Thickness; Dys-HDL: Dysfunctional high density lipoprotein; FHS: Framingham Heart Study; hsCRP: high sensitivity C-reactive protein; TGs: Triglycerides; IDF: International Diabetes Federation; LCAT: Lecithin: Cholesterol acyl transferase; LDL: Low density lipoprotein; Lp[a]: Lipoprotein [a]; MS: Metabolic syndrome NCEP ATP III: National Cholesterol Educational Program ATP III; NIH: National Institutes of Health; RCT: Reverse cholesterol transport; SAIs: South Asian immigrants; T2D: Type II diabetes; WC: Waist circumference; WHO: World Health Organization

## Conflict of interests

The authors declare that they have no competing interests.

## Authors' contributions

All authors have read and approved the final manuscript

**SD**= conceived this study, secured NIH funding, finished the study and wrote the paper

**MB and RH**= performed DNA isolation, sequencing and analysis, proof read paper and made changes

**JV and KG**= reviewed blinded IMT reports, provided results, supervised ultrasound technicians, proof read paper and made changes

**JW and LD**= performed all analyses under the direction of the PI, proof read paper and made changes

## Appendix 1

(Table 7)

**Table 7:** Definition of Metabolic Syndrome (MS)

	IDF (2005)	NCEP ATP III (2001)	European Group (1999)	WHO 1999
	Central obesity (ethnicity-specific)‡ and *two *or more of the following:	*Three *or more of the following:	Non-diabetics with insulin resistance§ and *two *or more of the following:	T2 D, impaired glucose tolerance, impaired fasting glucose, or insulin resistance* plus *two *or more of the following:

**Glucose (fasting)**	≥100 mg/dL (5.6 mmol/L) or T2D	≥110 mg/dL (6.1 mmol/L)†	≥110 mg/dL (6.1 mmol/L), but non-diabetic	

**Obesity**	Central obesity (ethnicity-specific)‡	Waist circumference ≥102 cm (40 in) in males or ≥ 88 cm (35 in) in females	Waist circumference ≥ 94 cm (37.0 in) in males or ≥ 80 cm (31.5 in) in females	Central obesity (WHR ≥ 0.90 in males or ≥ 0.85 in females) and/or BMI ≥ 30 kg/m2

**HDL Levels**	≥ 40 mg/dL (1.03 mol/L) in males, ≥ 50 mg/dL (1.29 mmol/L) in females, or treatment	≥ 40 mg/dL (1.03 mmol/L) in males or ≥ 50 mg/dL (1.29 mmol/L) in females	≥ 39 mg/dL (1.0 mmol/L) or treatment	≥ 35 mg/dL (0.9 mmol/L) in males or ≥ 39 mg/dL (≥ 1.0 mmol/L) in females

**Blood Pressure**	≥130/≥85 mmg or treatment	≥140/90 mm Hg or treatment	≥140/90 mm Hg or treatment	≥140/90 mm Hg

**Triglycerides**	≥150 mg/dL (1.7 mmol/L) or treatment	≥150 mg/dL (1.7 mmol/L)	≥150 mg/dL (1.7 mmol/L) or treatment	≥178 mg/dL (2.0 mmol/L) or treatment

*Refer to WHO publication for definitions of hyperglycemic states. Insulin resistance defined as glucose uptake below lowest quartile for background population under investigation, in hyperinsulinemic, euglycemic conditions. †Revised in 2004 to ≥100 mg/dL (5.6 mmol/L) to reflect the American Diabetes Association's updated definition of

impaired fasting glucose.9,132 ‡Defined as ≥ 94 cm (males) or ≥ 0 cm (females) in Europids and ≥ 90 cm (males) or ≥ 80 cm (females) in South Asians, among others. §Defined as the 25% of the non diabetic population with the highest insulin resistance or the highest fasting insulin concentrations.

**Abbreviations: **BMI, body mass index; European group = European Group for the Study of Insulin Resistance; HDL, high-density lipoprotein; IDF, International Diabetes Federation; NCEP

ATP III, National Cholesterol Education Program Adult Treatment Panel; T2 D, type II diabetes mellitus; WHO, World Health Organization; WHR, waist-to-hip ratio.
